# Social media consumption and depressive symptoms during the COVID-19 lockdown: the mediating effect of physical activity

**DOI:** 10.3389/fpsyt.2023.1120230

**Published:** 2023-12-07

**Authors:** Amy Chan Hyung Kim, James Du, Damon P. S. Andrew

**Affiliations:** Department of Sport Management, Center for Sport, Health, and Equitable Development (cSHED), Florida State University, Tallahassee, FL, United States

**Keywords:** social media consumption, depressive symptoms, physical activity, COVID-19, social media, mental health

## Abstract

**Introduction:**

Social media platforms played a critical role during the COVID-19 pandemic. This study aimed to explore: (1) the changes in social media consumption patterns, physical activity levels/sedentary behavior, and depressive symptoms, and (2) how the changes in social media consumption patterns predict the changes in depressive symptoms while investigating the mediating role of changes in physical activity levels/sedentary behavior between before, and after the COVID-19 lockdown among U.S. adults with different age clusters.

**Methods:**

A total of 695 U.S. participants completed an online questionnaire via MTurk, and participants were asked to recall their social media consumption patterns, physical activity/sedentary behavior, depressive symptoms in January and May of 2020 while covariates included non-physical activity health behavior including diet quality, alcohol consumption, smoking, and sleep quality.

**Results:**

The results of Bayesian significance testing of changes showed that the older participants tended to spend more time with content-focused social media platforms during the lockdown. While significantly increased sitting time was reported by all age clusters, no significant changes were found in activity levels. Additionally, the middle-aged and older participants reported significantly higher depressive symptoms. The findings of a multigroup structural analysis showed the significant mediating effect of moderate-to-vigorous physical activity on the relationship between changes in social media consumption and depressive symptoms.

**Discussion:**

This study highlights the need for targeting specific social media platforms for older adults and the importance of moderate-to-vigorous physical activity to alleviate the mental health issues resulting from social media consumption. The result of this study also highlights the need for sport-based intervention programs in the future and the need for more social media campaigns at the institution/organization levels established by public health stakeholders and policy makers to promote physical activity and maximize population perception and reach during the pandemic.

## 1 Introduction

Social media platforms played a critical role during the COVID-19 pandemic. As a consistently available communication tool, various information was generated, disseminated, and consumed via different types of social media platforms (1). Each social media platform served as a source for up-to-date information on progress regarding the pandemic and for entertainment while non-pharmaceutical interventions (e.g., shelter-in-place policies or stay-at-home-orders) were enacted. Since the COVID-19 outbreak, increases in social media use have been reported across the world – roughly 4.55 billion users in October 2021, compared to 3.8 billion users in January 2020. Notably, North America has the highest social media saturation rates (2, 3). Moreover, it was reported that there was a significant spike in the average time spent on social media among U.S. users in 2021, with 65 min daily compared to 54 min daily in 2019 (4).

The effects of social media consumption on mental health outcomes are well-known as a double-edged sword. During the pandemic, several studies of adults showed that increased social media consumption was related to lower loneliness (3), higher psychological well-being and happiness (2). Conversely, some studies found that increased social media consumption was associated with higher odds of anxiety (5), higher depression (5–7), higher psychosocial distress (8), higher loneliness (9), decreased life satisfaction (10), and overall poorer mental health (11). While these contradicting studies were consistent in their exploration of the direct relationships between social media consumption and various mental health outcomes, one of the most critical mediators, individual physical activity level, was overlooked.

It is well-known that the COVID-19-related lockdowns negatively impacted individual mood, feelings, and mental well-being (12). The COVID-19 lockdown also impacted patterns of physical activity, exercise, and sport participation due to closed facilities, gyms, and recreational centers (13). Interestingly, the findings of changes in physical activity during the pandemic have been mixed. An expected result has been a decrease in physical activity due to increased screen time resulted from increased use of smartphones, tablets, televisions, and video games, along with higher use of video chatting (14). Conversely, some studies found increased levels of physical activity resulting from more free time and home workout opportunities (14). In particular, social media platforms such as Twitter, Instagram, and TikTok have emerged as one type of technology-mediated means to promote the public’s active lifestyle even at home through collaborative fitness. The social platforms have facilitated user engagement by stimulating one’s social and hedonic values of active lifestyle (15). For instance, athletes all around the world posted positive videos promoting various types of fitness activity by using social campaigns such as “#fitnesschallenge,” “#plankchallenge,” or “#squatchallenge.” In addition, major sport organizations such as the International Olympic Committee and Australian Olympic Committee continued to engage with fans and participants by initiating social campaigns titled “train like an Olympian at home” (16).

We identified three major research gaps in the studies of changes in social media consumption, physical activity, and mental health after the COVID-19 outbreak. First, several studies investigated the direct relationship between changes in social media and mental health without considering the potential mediating role of physical activity on this relationship. For instance, while individual social media consumption may relate to decreased levels of physical activity, social media intervention studies confirmed that social media usage can positively change physical activity and health behaviors (17, 18). Thus, it is essential to take physical levels and sedentary behaviors into consideration to grasp the complete picture on the relationship between social media consumption and mental health.

We specified one’s physical activity level as a mediator based on the existing body of literature that social media can be an effective intervention to increase physical activity [e.g., (18)] and the therapeutic effect of physical activity participation on mental health [e.g., (19)]. Second, many studies in social media usage during the pandemic tended to disregard the unique nature of each platform by measuring social media usage as one construct instead of investigating each platform’s usage (20, 21). Moreover, most studies related to social media consumption during the pandemic investigated the limited types of social media platforms including Twitter (22), or WeChat (23), even though there are more types of platforms such as YouTube, TikTok, or Reddit. Notably, Masciantonio et al. (24) found different relationships between use of different social media platforms and well-being during the COVID-19 pandemic lockdown. For instance, while active Facebook usage and TikTok usage was not related to social support or satisfaction with life, active Instagram usage and Twitter usage related to satisfaction with life through positive social support. Yet, most social media and mental health studies either investigated the usage of one social media platform or categorized all media platforms as one category. Lastly, scholars have not considered the different population characteristics among adults – particularly age – when investigating the relationships among social media usage, physical activity, and mental health. Most of all, previous studies tended to focus on younger populations such as children, adolescents, or young adults when it comes to social media use, physical activity, and mental health (25–27). Additionally, as Fukukawa et al. (28) argued that age should be considered when examining the effect of physical activity on mental health among adult populations, there is a lack of research on the associations between social media use, physical activity, and mental health among different adult populations during the COVID-19 pandemic lockdown.

**Figure 1:**
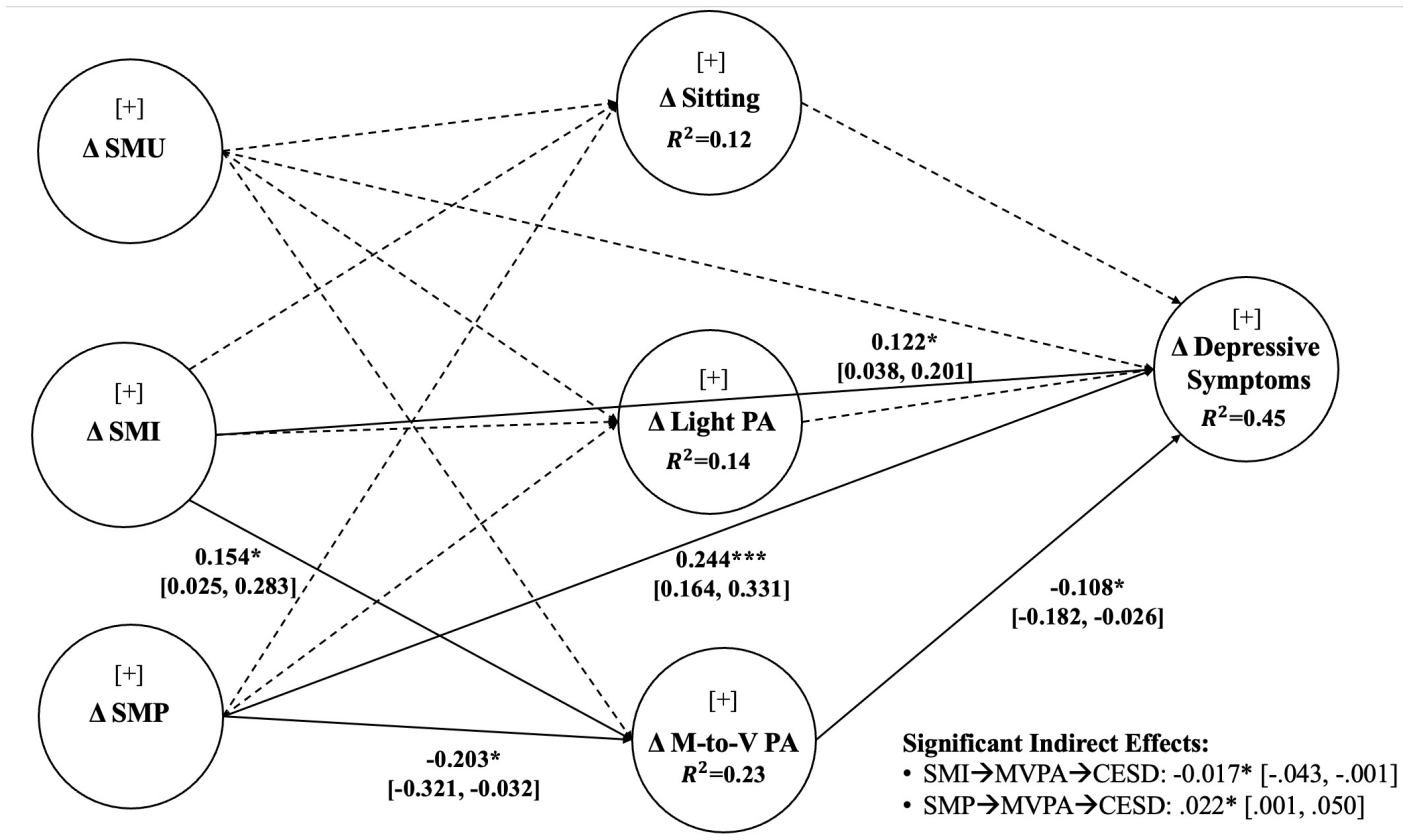
Research model.

In sum, to fill these gaps, this study’s primary research purposes are to (1) explore the changes in social media consumption patterns (i.e., social media usage, social media intensity, problematic social media usage, usage based on each social media platform), physical activity levels, and depressive symptoms and (2) investigate how the changes in social media consumption patterns (i.e., total social media usage, social media intensity, problematic social media usage) predict the changes in depressive symptoms while investigating the mediating role of changes in physical activity levels (i.e., sitting, light physical activity, moderate-to-vigorous physical activity) between before, and after the COVID-19 lockdown among different age clusters (i.e., young adults, middle-aged adults, older adults) (see Figure 1 for the proposed research model). Here, social media usage is defined as the duration and frequency of social media use (29), whereas social media intensity is defined as a social media user’s level of activity and engagement with social media (30). Problematic social media usage refers to a detrimental effect that occurs as a result of preoccupation and compulsion to excessive use in social media platforms (31). Among several mental illness indicators, we focused on depressive symptoms which is one of the most prevalent outcomes after experiencing traumatic events (32).

## 2 Materials and methods

### 2.1 Participants and study design

Participants were invited to recall their social media consumption, physical activity participation, and depressive symptoms in January 2020 and May 2020 via Amazon’s Mechanical Turk (MTurk). The platform has been useful as a data collection method for web-based research in health and medical research due to its higher reachability, higher reliability, and higher completion rate compared to conventional data collection methods such as paper-, telephone-, or in-person-based data capture approaches (33). In the present study, we employed a retrospective cross-sectional design. After obtaining consent, through Qualtrics, respondents answered screening questions about the respondent’s United States residency and age. As an attention question, we included “To continue with the survey”, please select “Somewhat agree” in the middle of the questionnaire. In the first week of June of 2020, the respondents were asked to recall their social media consumption, physical activity level, depressive symptoms, and non-health-behavior (i.e., diet quality, alcohol consumption, smoking, sleep quality) retrospectively at two timepoints: (1) January of 2020, which was immediately prior to the initial outbreak of the COVID-19 pandemic in the United States, and (2) May of 2020, which marked the peak of the first wave of infections and nationwide lockdowns (15). Three survey pools for young (18–39 years old), middle-aged (40–59 years old), and older (60 years and older) were created (34).

We used *a priori* Power Analysis to calculate the required sample size using the statsmodels in Python setting with the desired alpha at 0.05 and beta at 0.8 with an effect size equating to 0.5 (35) and the results yielded a minimum sample size of 695. Among 865 recorded responses who passed the screening questions, a total of 170 responses (55 young, 79 middle-aged, 36 older adults) were excluded because they failed to pass the attention question. In sum, a total of 695 responses (264 young, 234 middle-aged, 197 older adults) were included for further analysis. Survey respondents were from all states except for four states in the Midwest, including Montana, Wyoming, South Dakota, and Nebraska. Considering that COVID-19 least influenced these rural communities in the early period of the pandemic (36), the sample was reasonably representative for conducting the subsequent statistical analyses. This study protocol was approved by the Institutional Review Board at Florida State University (ID: STUDY00001406).

### 2.2 Measures

#### 2.2.1 Social media consumption pattern

For social media consumption pattern, we adopted three previously validated measures: social media usage [i.e., duration and frequency; (29)], social media intensity (30), and problematic social media use (31). For duration, we asked “Approximately how much time per day did you spend on social media for personal, non-work-related use in January 2020?,” whereas for frequency, we asked, “How often did you visit (social media platform name) in January 2020?” across twelve prominent social media platforms (i.e., Facebook, Instagram, Pinterest, LinkedIn, Twitter, Snapchat, YouTube, TikTok, WhatsApp, Reddit, Tumblr, and Vine). For social media intensity, a 6-item scale developed by Ellison et al. (30) was used. A sample item was “Social media was part of my everyday activity in January 2020” with a 5-point Likert scale from 1 = *strongly disagree* to 5 = *strongly agree*. For problematic social media use, we adopted a 6-item social media addiction scale originally developed by Andreassen et al. (31). A sample item was “How often did you spend a lot of time thinking about social media or planned use of social media in January 2020?” with a 5-point Likert scale from 1 = *very rarely* to 5 = *very often.* All questions included two sets of questions for January 2020 and May 2020, respectively.

#### 2.2.2 Physical activity

The International Physical Activity Questionnaire-Short Form (IPAQ-SF), a validated self-reported measurement tool for physical activity among various adult population surveys, was used (37). This 7-item questionnaire included the frequency and duration of four different levels of weekly physical activities: vigorous activity (such as heavy lifting or aerobics), moderate activity (such as doubles tennis), light activity (including walking), and sitting for January 2020 and May 2020, respectively.

#### 2.2.3 Depressive symptoms

To assess individual depressive symptoms, the 10-item Center for Epidemiologic Studies Depression Scale (CES-D-10) was employed. The reliability and validity of this scale has been consistently supported by previous large-scale survey studies (38, 39). The items reflect the respondents’ feelings and the respondents answered 10 items for both January 2020 and May 2020. A higher score indicates a higher level of depressive symptoms.

#### 2.2.4 Control variables

We included personal characteristics (i.e., age, marital status, household income, education, occupation, BMI, and Zip code) and non-physical-activity health behavior (i.e., diet quality, alcohol consumption, smoking, and sleep quality) as control variables. With acceptable validity and reliability, we used four items to assess one’s daily intakes of fruit and vegetables, weekly intakes of fast food and soft drinks (40). Alcohol consumption was evaluated by the validated 3-item AUDIT Alcohol Consumption Questions [AUDIT-C; (41)] including questions about frequency and intensity of regular drinking and heaving drinking. The validated 8-item Fagerstrom Test for Nicotine Dependence (FTND) was employed to assess one’s smoking status including frequency, amount, and dependency of smoking (42). The validated 3-item Pittsburg Sleep Quality Index (PSQI) was adopted to evaluate one’s sleep quality which includes average daily hours of sleep, overall sleep quality, and sleep latency (43). Similar to social media consumption patterns, physical activity levels, and depressive symptoms, the respondents provided answers for two sets of questions for both the January 2020 and May 2020 timeframe.

### 2.3 Analyses

First, for testing significant changes in social media consumption patterns, physical activity, and depressive symptoms in each age group, Bayesian significance testing of changes was employed using the R2WinBUGS package in R (44). More specifically, we used the mean and standard deviation of the data recalled in January 2020 to specify distributions of informative priors in our Bayesian analysis. Next, we defined the precision of a normal distribution as the inverse of the squared standard deviation, where we assumed that the observations at both time points derive from normal distributions with the same precision tau. Then, we specified parameters for running the Markov Chain Monte Carlo (MCMC) simulation in R2WinBUGS to ensure the chain convergence was established for the estimated posterior distributions, with the number of chains equating to 10 and the total number of MCMC iterations per chain set at 20,000, while discarding the first 1,000 iterations as burn-in. The Bayesian approach was used due to its tendency to be less sensitive to the influence associated with missing values and asymptotic assumptions. Subsequently, a multigroup structural analysis was employed to evaluate the global empirical model within the component/variance-based Structural Equation Modeling framework using SmartPLS 4.0.

## 3 Results

Table 1 shows the descriptive statistics of respondents, and the baseline averages of the included parameters are shown in Table 2. While roughly 60% of respondents were male, most participants were Caucasian (70.2%), well-educated (74.8% with a 4-year college or more advanced degrees) and lived in a somewhat middle-class family with an annual household income between $25,000 and $75,000 as of 2019. Notably, the demographic breakdown was mostly consistent across three different age groups, except that more female respondents (65%) were identified in the older adult group.

**Table 1:** Descriptive statistics of sample demographic profiles.

Age groups	Parameter	Mean/Mode^†^	Frequency	Percent
Global sample (*n* = 695)	Age	45.85	n/a	n/a
	Std. deviation of age	15.42	n/a	n/a
	Ethnicity	Caucasian	488	70.20%
	Gender	Male	417	60.00%
	Education	4-Year College & Advanced Degrees	520	74.80%
	Income	$25,000 to $75,000	429	61.80%
	Job	Employed (Full-time + part-time)	570	82.00%
Young (18–39) (*n* = 264)	Age	29.78	n/a	n/a
	Std. deviation of age	4.08	n/a	n/a
	Ethnicity	Caucasian	161	61.00%
	Gender	Male	189	71.60%
	Education	4-Year College & Advanced Degrees	219	82.90%
	Income	$25,000 to $75,000	159	60.30%
	Job	Employed (Full-time + part-time)	253	95.80%
Middle aged (40–59) (*n* = 234)	Age	46.74	n/a	n/a
	Std. deviation of age	4.93	n/a	n/a
	Ethnicity	Caucasian	152	65.00%
	Gender	Male	158	67.50%
	Education	4-Year College & Advanced Degrees	193	82.40%
	Income	$25,000 to $75,000	154	65.80%
	Job	Employed (Full-time + part-time)	217	92.80%
Old (60+) (*n* = 197)	Age	66.00	n/a	n/a
	Std. deviation of age	4.50	n/a	n/a
	Ethnicity	Caucasian	175	88.80%
	Gender	Female	127	64.50%
	Education	4-Year College & Advanced Degrees	108	54.80%
	Income	$25,000 to $75,000	116	58.90%
	Job	Employed (Full-time + part-time)	100	50.80%

^†^Means were reported for continuous variables, and modes were displayed for ordinal (e.g., income) or categorical variables (e.g., gender); n/a = not applicable.

**Table 2:** The significance of changes by age groups using Bayesian analysis.

Age groups	Parameter	Baseline mean in January	Posterior			95% CI	
			Mean	S.D.	*p* (2-tailed)	Lower bound	Upper bound
Global sample (*n* = 695)	Δ_LPA	2.30	−0.084	1.932	0.251	−0.228	0.060
	Δ_MVPA	5.32	−0.060	3.258	0.626	−0.303	0.183
	Δ_SPA***	5.26	0.432	2.384	0.000	0.255	0.610
	Δ_CESD***	11.62	1.186	4.608	0.000	0.842	1.529
	Δ_SMU***	3.46	1.492	3.055	0.000	1.264	1.720
	Δ_SMP***	2.76	0.208	0.640	0.000	0.160	0.256
	Δ_SMI***	3.42	0.190	0.691	0.000	0.139	0.242
	Δ_Meta***	4.74	0.288	1.431	0.000	0.181	0.394
	Δ_Instagram***	3.95	0.355	1.405	0.000	0.251	0.460
	Δ_Pinterest***	3.38	0.279	1.372	0.000	0.177	0.381
	Δ_LinkedIn***	3.33	0.233	1.327	0.000	0.134	0.332
	Δ_Twitter***	3.93	0.292	1.484	0.000	0.181	0.403
	Δ_Snapchat***	3.10	0.335	1.396	0.000	0.231	0.439
	Δ_YouTube***	4.48	0.345	1.440	0.000	0.238	0.453
	Δ_TikTok***	3.17	0.413	1.489	0.000	0.302	0.524
	Δ_WhatsApp***	3.69	0.206	1.308	0.000	0.108	0.303
	Δ_Reddit***	3.36	0.183	1.342	0.000	0.083	0.283
	Δ_Tumblr***	2.95	0.199	1.289	0.000	0.102	0.295
	Δ_Vine**	2.98	0.180	1.408	0.001	0.075	0.285
Young (18–39) (*n* = 264)	Δ_LPA	2.89	−0.186	1.944	0.122	−0.422	0.051
	Δ_MVPA	6.05	0.172	3.134	0.372	−0.209	0.554
	Δ_SPA***	4.62	0.713	2.619	0.000	0.394	1.031
	Δ_CESD	15.15	0.318	4.354	0.236	−0.211	0.848
	Δ_SMU***	4.35	2.225	3.802	0.000	1.763	2.688
	Δ_SMP***	3.23	0.165	0.682	0.000	0.082	0.248
	Δ_SMI**	3.53	0.132	0.702	0.003	0.047	0.217
	Δ_Meta**	4.86	0.280	1.539	0.003	0.093	0.468
	Δ_Instagram***	4.71	0.424	1.568	0.000	0.234	0.615
	Δ_Pinterest**	3.98	0.318	1.607	0.001	0.123	0.514
	Δ_LinkedIn*	4.12	0.201	1.604	0.043	0.006	0.396
	Δ_Twitter	4.66	0.106	1.690	0.309	−0.100	0.312
	Δ_Snapchat***	4.00	0.417	1.693	0.000	0.211	0.623
	Δ_YouTube**	4.93	0.341	1.579	0.001	0.149	0.533
	Δ_TikTok***	4.16	0.417	1.613	0.000	0.220	0.613
	Δ_WhatsApp**	4.78	0.250	1.466	0.006	0.072	0.428
	Δ_Reddit*	4.13	0.227	1.543	0.017	0.040	0.415
	Δ_Tumblr	3.86	0.152	1.523	0.107	−0.034	0.337
	Δ_Vine	3.88	0.129	1.672	0.212	−0.075	0.332
Middle aged (40–59) (*n* = 234)	Δ_LPA	2.51	−0.048	2.121	0.728	−0.323	0.226
	Δ_MVPA	6.06	−0.369	3.723	0.131	−0.850	0.113
	Δ_SPA	4.88	0.202	2.451	0.209	−0.115	0.519
	Δ_CESD**	13.19	0.915	4.923	0.005	0.278	1.551
	Δ_SMU***	3.77	1.491	2.922	0.000	1.113	1.869
	Δ_SMP***	3.12	0.286	0.741	0.000	0.190	0.381
	Δ_SMI***	3.55	0.266	0.775	0.000	0.166	0.367
	Δ_Meta**	4.85	0.325	1.538	0.001	0.126	0.524
	Δ_Instagram***	4.50	0.479	1.578	0.000	0.274	0.683
	Δ_Pinterest***	3.88	0.410	1.483	0.000	0.218	0.602
	Δ_LinkedIn***	3.86	0.449	1.414	0.000	0.266	0.632
	Δ_Twitter***	4.32	0.517	1.624	0.000	0.307	0.727
	Δ_Snapchat***	3.61	0.491	1.495	0.000	0.298	0.685
	Δ_YouTube**	4.79	0.286	1.628	0.008	0.076	0.497
	Δ_TikTok***	3.74	0.611	1.725	0.000	0.388	0.834
	Δ_WhatsApp**	4.40	0.312	1.594	0.003	0.106	0.518
	Δ_Reddit*	3.88	0.252	1.564	0.014	0.050	0.454
	Δ_Tumblr***	3.44	0.389	1.464	0.000	0.199	0.578
	Δ_Vine***	3.50	0.402	1.589	0.000	0.196	0.607
Old (60+) (*n* = 197)	Δ_LPA	1.28	0.009	1.662	0.940	−0.226	0.244
	Δ_MVPA	3.47	−0.006	2.783	0.976	−0.399	0.387
	Δ_SPA*	6.55	0.331	1.896	0.015	0.063	0.598
	Δ_CESD***	5.01	2.670	4.198	0.000	2.077	3.263
	Δ_SMU***	1.91	0.511	1.344	0.000	0.321	0.701
	Δ_SMP***	1.71	0.173	0.404	0.000	0.116	0.230
	Δ_SMI***	3.12	0.178	0.551	0.000	0.100	0.255
	Δ_Meta**	4.45	0.254	1.119	0.002	0.096	0.412
	Δ_Instagram*	2.26	0.117	0.803	0.043	0.003	0.230
	Δ_Pinterest	1.96	0.071	0.718	0.166	−0.030	0.172
	Δ_LinkedIn	1.62	0.020	0.553	0.607	−0.058	0.098
	Δ_Twitter***	2.51	0.274	0.849	0.000	0.154	0.394
	Δ_Snapchat	1.27	0.041	0.523	0.277	−0.033	0.115
	Δ_YouTube***	3.52	0.421	0.915	0.000	0.292	0.551
	Δ_TikTok**	1.18	0.173	0.846	0.005	0.053	0.292
	Δ_WhatsApp	1.38	0.020	0.319	0.372	−0.025	0.065
	Δ_Reddit	1.72	0.041	0.493	0.249	−0.029	0.110
	Δ_Tumblr	1.14	0.036	0.383	0.194	−0.019	0.090
	Δ_Vine	1.15	−0.015	0.410	0.603	−0.073	0.043

Monte Carlo Sampling Seed: 200,000. CI = Bayesian 95% credible intervals; LPA, light intensity physical activity; MVPA, moderate and vigorous-intensity physical activity; SMU, social media usage; SMI, social media intensity; SMP, social media addiction; SPA, sitting; *p < 0.05; **p < 0.01; ***p < 0.001.

The global results of the Bayesian significance testing of changes showed that social media usage, including individual’s usage frequency in hours (Δμ = 1.49, 95% CI [1.26, 1.72]), problematic consumption (Δμ = 0.21, 95% CI [0.16, 0.26]), and usage intensity (Δμ = 0.19, 95% CI [0.14, 0.24]), significantly increased among the respondents between January 2020 and May 2020 across all social media platforms (See Table 2). The greatest increase was observed in the usage of short-video platform TikTok (Δμ = 0.41, 95% CI [0.30, 0.52]) while behavioral engagement in community-based Reddit (Δμ = 0.18, 95% CI [0.08, 0.28]) experienced the least increase. Significant changes in social media usage were not universally applicable to all age segments; older adults showed a relatively small increase across the most social media platforms except for Meta, Instagram, Twitter, YouTube, and TikTok (see Table 2). Middle-aged adults showed an increase in widest range of social media platforms (all twelve social media platforms), whereas young adults showed significant increases in all but Twitter, Tumblr, and Vine. In terms of physical activity levels, despite slight decreases of hours in vigorous, moderate, and light physical activity (walking) globally, the changes were not statistically significant. Interestingly, except for the middle-aged respondents, young adults (Δ_SPA_ = 0.712, *p* < 0.001, 95% CI [0.394, 1.031]) and older adults (Δ_SPA_T = 0.331, *p* < 0.05, 95% CI [0.063, 0.598]) had a significantly increased sitting time, indicating an increased level of sedentary behavior during the COVID-19 lockdown. When it comes to depressive symptoms, there were no significant changes among young participants, whereas middle-aged (Δ_CESD_Total = 0.915, *p* < 0.01, 95% CI [0.278, 1.551]) and older participants (Δ_CESD_Total = 2.670, *p* < 0.001, 95% CI [2.077, 3.263]) had significantly higher levels of depressive symptoms after the lockdown (see Table 2 for further information) (see Table 3).

**Table 3:** The results of structural analysis.

Paths	Bootstrapping sample mean	STDEV	T statistics	*p* values	Bootstrapping 95% CI	
Direct relationships						
Δ_LPA → Δ_CESD	−0.047	0.038	1.341	0.180	−0.120	0.008
Δ_MVPA → Δ_CESD*	−0.108	0.055	1.971	0.049	−0.182	−0.026
Δ_SMC → Δ_CESD	−0.001	0.042	0.155	0.877	−0.086	0.057
Δ_SMC → Δ_LPA	−0.016	0.087	0.265	0.791	−0.167	0.111
Δ_SMC → Δ_MVPA	−0.014	0.062	0.129	0.897	−0.101	0.104
Δ_SMC → Δ_SPA	0.049	0.061	0.796	0.426	−0.054	0.147
Δ_SMI → Δ_CESD*	0.122	0.050	2.446	0.015	0.038	0.201
Δ_SMI → Δ_LPA	0.063	0.074	0.986	0.324	−0.046	0.191
Δ_SMI → Δ_MVPA*	0.154	0.081	2.029	0.043	0.025	0.283
Δ_SMI → Δ_SPA	−0.055	0.070	0.830	0.406	−0.189	0.045
Δ_SMP → Δ_CESD***	0.244	0.051	4.859	0.000	0.164	0.331
Δ_SMP → Δ_LPA	−0.007	0.067	0.090	0.929	−0.120	0.104
Δ_SMP → Δ_MVPA*	−0.203	0.086	2.401	0.016	−0.321	−0.032
Δ_SMP → Δ_SPA	0.098	0.062	1.587	0.113	−0.007	0.197
Δ_SMU → Δ_CESD	−0.001	0.023	0.007	0.994	−0.037	0.040
Δ_SMU → Δ_LPA	−0.014	0.044	0.359	0.719	−0.088	0.057
Δ_SMU → Δ_MVPA	−0.008	0.049	0.204	0.838	−0.082	0.074
Δ_SMU → Δ_SPA	0.091	0.053	1.708	0.088	0.001	0.176
Δ_SPA → Δ_CESD	0.048	0.039	1.274	0.203	−0.013	0.113
Controls → Δ_CESD*	−0.270	0.136	1.981	0.048	−0.361	−0.011
Indirect relationships						
Δ_SMP → Δ_MVPA→Δ_CESD*	0.022	0.011	1.969	0.049	0.001	0.050
Δ_SMP → Δ_SPA→Δ_CESD	0.005	0.006	0.886	0.376	−0.009	0.020
Δ_SMU → Δ_MVPA→Δ_CESD	0.002	0.005	0.207	0.836	−0.007	0.011
Δ_SMU → Δ_LPA → Δ_CESD	0.001	0.003	0.310	0.757	−0.002	0.007
Δ_SMI → Δ_MVPA→Δ_CESD*	−0.017	0.009	1.966	0.050	−0.043	−0.001
Δ_SMC → Δ_MVPA→Δ_CESD	0.001	0.008	0.116	0.908	−0.011	0.014
Δ_SMC → Δ_SPA→Δ_CESD	0.003	0.004	0.540	0.589	−0.001	0.015
Δ_SMU → Δ_SPA→Δ_CESD	0.004	0.005	0.930	0.353	0.000	0.017
Δ_SMC → Δ_LPA → Δ_CESD	0.001	0.005	0.235	0.814	−0.004	0.015
Δ_SMI → Δ_LPA → Δ_CESD	−0.004	0.005	0.745	0.456	−0.019	0.001
Δ_SMI → Δ_SPA→Δ_CESD	−0.003	0.005	0.577	0.564	−0.019	0.001
Δ_SMP → Δ_LPA → Δ_CESD	0.000	0.004	0.079	0.937	−0.005	0.007

CI = 95% biased-corrected confidence intervals; CESD, depressive symptoms; MVPA, moderate and vigorous-intensity physical activity; LPA, light intensity physical activity; SMU, social media usage; SMI, social media intensity; SMP, social media addiction; SPA, sitting; *p < 0.05, ***p < 0.001.

We also created a series of interaction terms between age groups and all major variables to test if changes in behavioral outcomes and depressive symptoms varied by age cohorts. The findings indicated that changes in social media usage differed significantly between young adults and older adults (*p* < 0.001), as well as between middle-aged adults and older adults (*p* < 0.01). Additionally, changes in depressive symptoms differed significantly between young adults and older adults (*p* < 0.001) and between middle-aged and older adults (*p* < 0.001). Notably, changes in self-reported use of different social media platforms significantly differed between middle-aged adults and older adults in terms of Instagram (*p* < 0.05), Pinterest (*p* < 0.05), LinkedIn (*p* < 0.01), TikTok (*p* < 0.01), Tumblr (*p* < 0.05), and Vine (*p* < 0.01). Finally, changes in social media intensity, problematic social media use, and reported physical activity were not significantly different by age.

The results of the multigroup structural analysis (see Figure 1) showed a significant increase in social media intensity (*β* = 0.122, *p* < 0.05, 95% CI [0.038, 0.201]) and problematic social media usage (*β* = 0.244, *p* < 0.001, 95% CI [0.164, 0.331]) significantly led to a heightened level of depressive symptoms. Moderate-to-vigorous physical activity levels mediated both the relationship between social media intensity and depressive symptoms (*β* = −0.017, *p* < 0.05, 95% CI [−0.043, −0.001]) and between problematic social media consumption and depressive symptoms (*β* = 0.022, *p* < 0.05, 95% CI [0.001, 0.050]). That is, moderate and vigorous physical activity, such as participation in active sports, could alleviate the adverse effect of increased depressive symptoms resulting from increased social media consumption and addictive social media behaviors. No significant heterogeneity effect across different age cohorts were found (see Table 4) except for one path between social media intensity and depressive symptoms. The younger adults inclined to have a stronger relationship between social media intensity and depressive symptoms compared to older adults and middle-aged adults (*β* = 0.232, *p* = 0.049). No difference was found between middle-aged adults and older adults.

**Table 4:** Significant results of multigroup comparisons.

Path of hypothesis	Welch-Satterthwaite (W-S) significant testing†		
	*Diff* |Y-O|	*Diff* |Y-M|	*Diff |M-O|*
ΔSMI → ΔCESD	0.182*	0.100*	0.081 (n.s.)
	1.979	1.972	0.721

CESD, depressive symptoms, SMI, social media intensity, M, middle aged cohort, O, older cohort, Y, younger cohort; * *p* < 0.05, n.s. stands for non-significant. †The W-S test assumes unequal variances across age clusters. T-statistics of the W-S test were displayed for path differences.

## 4 Discussion

The first purpose of this study was to explore changes in social media consumption patterns, physical activity levels, and depressive symptoms between before, and after the COVID-19 lockdown among young adults, middle-aged adults, and older adults. Regarding social media usage, older adults tended to spend more time with Instagram, Twitter, YouTube, and TikTok, which are content-focused social media platforms, whereas no significant changes were found in use of message-based platforms such as Snapchat or WhatsApp during the COVID-19 lockdown. Notably, middle-aged adults showed the most significant changes in use of social media platforms, spending more time with all twelve social media platforms. Younger respondents also spent more time on the wide range of social media after the COVID-19 lockdown, reporting increased use of all platforms but Twitter, Tumblr, and Vine. Yet, it should be noted that the baseline Twitter usage was relatively high. The findings implied that specific social media platforms should be targeted to increase reach to older adults in particular.

In terms of physical activity levels, while significantly increased sitting time was reported by all respondents regardless of age, no significant change was found in activity levels. This finding highlights the importance of effective social media campaigns to promote physical activity during the pandemic. For instance, during the COVID-19, two physician athletes of the United States initiated a social media campaign to promote physical activity among the general population with the #SocialDistancingFitnessChallenge (45). In March and April of 2020, these physicians posted a 5-day workweek and received positive feedback from social media users that those users engaged in physical activity during that time inspired by the posts (45). In spite of various types of individual-level campaigns during this time, organization-level campaigns have been lacking. For instance, between March 12 of 2020 (i.e., the day President Trump declared a national emergency concerning the COVID-19 outbreak) and December 2021, only 73 out of 4,137 postings (roughly 1.8%) of the Centers for Disease Control and Prevention (CDC) and only 160 postings out of 7,175 postings (roughly 2.2%) of the World Health Organization (WHO) were about physical activity. At the organization/institution levels, more social-media-based campaigns for physical activity may need to be designed and implemented to stimulate more active lifestyles regardless of the age of populations. In fact, the University of Milan conducted a social-media-based physical activity promotion campaign with “#StayHomeStayFit” providing useful general information and credible suggestions regarding physical activity and psychological support for the general population during the COVID-19 lockdown, attracting massive attention according to page views and reactions (46). This type of social media campaign, if established by public health stakeholders, policymakers and institutions, would maximize population perception and reach during the pandemic.

The significantly increased sitting time also indicates reducing sedentary behavior is critical. As Manini et al. (47) argued, interventions targeting sedentary behavior are distinctive from targeting physical activity. In particular, it is essential to consider a life course perspective that assumes sedentary behavior is age and life stage dependent. In this context, the importance of addressing environmental factors has been highlighted to reduce sedentary behavior. To fight against increased sedentary behavior during the COVID-19 lockdown, promoting environment change to reduce sedentary behavior seems critical. For instance, using new technology (e.g., standing desk, desk treadmills), new workspace ideas (e.g., active workstations at home), and developing specific policies (e.g., break times) might be effective interventions.

Consistent with expectations, the middle-aged and older respondents reported significant higher depressive symptoms, whereas no significant change was found among younger respondents. Nevertheless, it should be noted that the baseline of older respondents’ depressive symptom was very low, while middle-aged (CESD_Total = 13.19) and younger adults (CESD_Total = 15.15) showed already relatively higher levels of depressive symptoms in January 2020. While this study solely focused on the negative mental health state, future studies may also need to investigate positive states such as happiness, optimism, or purpose in life.

Even though this study focused on social media use as an antecedent of mental health, it should be noted that some studies conducted in Italy contended that pre-existing mental issues could result in excessive and problematic social media use (48) among the Italian population. Sampogna et al. (48) found that people with mental disorders tended to consume significantly more hours on social media compared to the general population in Italy during the COVID-19 pandemic lockdown. Similarly, Volpe et al. (49) reported that general psychopathology, stress, anxiety, depression, and social isolation played a significant role on problematic social media use along with video gaming and internet use. Further studies with US adults, specifically patients with pre-existing mental health issues or disorders, may be beneficial to explore the nature of this relationship between social media consumption and mental health further.

The second purpose of this study aimed to investigate the mediating effect of changes in different levels of physical activity on the relationship between the changes in social media consumption and depressive symptoms during the COVID-19 lockdown. The findings highlight the importance of moderate-to-vigorous physical activity (MVPA; e.g., playing doubles tennis, running, cycling), implying potential differential effects compared to light physical activity (e.g., walking) on the relationship between media consumption and depressive symptoms. While some prior studies found that even light-intensity physical activity has positive mental health effects [e.g., (50)], some studies targeting the relief of depression via endorphin secretion is only associated with MVPA [e.g., (51)]. The findings of this study suggest that MVPA tended to alleviate the increased levels of depressive symptoms linked with the increased intensity of social media consumption and increased problematic social media behavior. Therefore, MVPA could be a potential coping strategy that can ease depressive symptoms resulting from excessive social media consumption. Considering the levels of MVPA was a significant mediator, further prospective and experimental studies would be helpful to examine what types of MVPA would be most effective on alleviating the mental health issues resulting from social media consumption.

When it comes to physical activity, there are three different types: physical activity [i.e., “bodily movement produced by skeletal muscles that results in energy expenditure,” (52), p. 126], exercise [i.e., “physical activity that is planned, structured, repetitive, and purposive in the sense that improvement or maintenance of one or more components of physical fitness is an objective,” (52), p. 128], and sport [i.e., “all forms of physical activity which, through casual or organized participation, aim at expressing or improving physical fitness and mental wellbeing, forming social relationships or obtaining results in competition at all levels,” (53)]. Unlike unorganized physical activity, such as gardening or dog walking, and exercise, such as muscular strength training, sport is capable of achieving a result requiring physical exertion and/or physical skill, which, by its nature, is competitive and social (54). In fact, previous research supports the notion that the mental health effects of exercise may be different than that of sport. For instance, while Krogh et al. (55) concluded that the effects of exercise were insignificant on mental health outcomes, Asztalos et al. (56) concluded that only sport participation and no other type of physical activity was consistently related to lower stress and distress because sport is the only form of MVPA that aims for enjoyment and social interactions compared to other types such as exercise or biking to work. Therefore, further research may need to investigate the potential different effects of unorganized physical activity, exercise, and sport on the relationship between social media consumption and depressive symptoms.

Even though this study focused on the intensity and addictive behavior of social media consumption in general, different types of social media platforms were not considered. For instance, the addictive behavior with YouTube may have different effects on physical activity levels compared to the addictive behavior with Reddit. The type of consumed contents also should be considered. For example, consuming fitness-related content may have different effects than consuming food-related content. Lastly, the purpose of social media consumption also should be considered. Social media consumption can play various roles in one’s life: information seeking, social networking, business transactions, and so forth. In particular, using social media platforms for social purposes (e.g., social support, social relationships) have been known to be beneficial for one’s mental health during challenging times (57).

## 5 Limitations

The present study had several limitations that have implications for future research. First, our study should not be generalized to dissimilar populations considering our convenience samples featured a relatively healthy population with high levels of physical activity, low levels of depressive symptoms and an average or higher socioeconomic status. Moreover, the participants recalled their behavior 5 months following the first targeted recall date of January of 2020, which may result in recall bias. Though COVID-19 has been an influential global event that might prompt stronger recall of behaviors and moods connected to the pandemic, it is important to note that the present study was not immune from potential recall bias.

It is also should be noted that IPAQ does not differentiate between the different domains of physical activity such as work-related physical activity, household physical activity, leisure-time physical activity. Considering leisure-time physical activity has been recognized as a more significant predictor of mental health outcomes (58), the use of IPAQ might not capture the role of different types of physical activity. Additionally, this study did not consider the different types of social media usage. For instance, an active usage such as interacting directly with others through posting new content or adding comments to other posts can be distinguished from a passive usage such as reading, and skimming the content and posts of others. Some studies found that active social media usage tended to positively associate with one’s well-being, whereas passive usage inclined to negatively associated with well-being (24, 59, 60). Thus, future research may need to consider the different types of social media usage when it comes to one’s mental health and physical activity participation. Additionally, even though depressive symptoms have been identified as one of the significant mental health indicators related to traumatic events, there are other critical indicators such as anxiety or stress. These mental health indicators also need to be examined in the future research. Lastly, the present study examined only one outcome, depressive symptoms, which presents the negative state of one’s emotions. More diversified psychological outcomes, including positive states such as positive psychological well-being (e.g., optimism, happiness, and contentment), should be investigated in the future.

## Data availability statement

The raw data supporting the conclusions of this article will be made available by the authors, without undue reservation.

## Ethics statement

The studies involving humans were approved by Florida State University Office of Research. The studies were conducted in accordance with the local legislation and institutional requirements. The participants provided their written informed consent to participate in this study.

## Author contributions

AK, JD, and DA contributed to the conception and design of the study and wrote sections of the manuscript. AK and DA organized the data collection. JD performed the statistical analyses. All authors contributed to the article and approved the submitted version.
